# Metabolic rate and saliva cortisol concentrations in socially housed adolescent guinea pigs

**DOI:** 10.1007/s00360-024-01576-y

**Published:** 2024-07-17

**Authors:** Matthias Nemeth, Susanna Fritscher, Klara Füreder, Bernard Wallner, Eva Millesi

**Affiliations:** 1 Department of Behavioral and Cognitive Biology, Faculty of Life Sciences, University of Vienna, University of Vienna Biology Building, Djerassiplatz 1, 1030 Vienna, Austria; 2 University Research Platform “The Stress of Life (SOLE)”, University of Vienna, Vienna, Austria

**Keywords:** Metabolic rate, Oxygen consumption, Saliva cortisol, Adolescence, Sex difference, Repeatability

## Abstract

An individual’s energetic demands and hence metabolic rate can strongly change during adolescence, a phase characterized by profound morphological, physiological, and endocrine changes. Glucocorticoid hormones (e.g. cortisol) are released in response to hypothalamic–pituitary–adrenal–axis activity, modulate several metabolic processes, and can also be linked to increased metabolic rate. In domestic guinea pigs (*Cavia aperea f. porcellus*) housed in same-sex groups, cortisol concentrations increase during adolescence in males but remain stable in females, which was suggested to be related to different energetic demands by age. We therefore measured metabolic rate through oxygen (O_2_) consumption over 2.5 h in male and female guinea pigs housed in same-sex groups during adolescence at ages of 60, 120, and 180 days, which was paralleled by analyses of saliva cortisol concentrations before and after the measurement. The statistical analyses involved whole body metabolic rate (ml O_2_/h), body mass-corrected metabolic rate (ml O_2_/h/kg), and body mass-independent metabolic rate (ml O_2_/h statistically corrected for body mass). We found increasing cortisol concentrations with age in males only, but none of the three metabolic rate analyses revealed a sex difference by age. On the individual level, repeatability across ages was found in metabolic rate as well as in body mass and cortisol concentrations after the measurement, but not in “basal” cortisol concentrations. Our results suggest no sex-specific changes in metabolic rate and hence equal energetic demands in male and female guinea pigs during adolescence. Moreover, metabolic rate clearly represents a highly stable physiological trait already early in a guinea pig’s life irrespective of rather fluctuating cortisol concentrations.

**Supplementary Information:**

The online version contains supplementary material available at 10.1007/s00360-024-01576-y.

## Introduction

The structural and functional changes associated with ontogeny cause different energetic requirements (Hou et al. 2008) and can represent profound challenges for any organism. Measurements of metabolic rate (including basal or resting metabolic rate), e.g. through an individual’s oxygen consumption, can provide valuable information in this context. Metabolic rate is known to be positively linked to body mass and individual growth rates (Careau et al. 2013), but the metabolic costs associated with growth are strongly suggested to change over time (Vézina et al. 2009). Individual differences in body composition, including lean body mass and organ size, are considered to play a critical role (Vézina et al. 2009; Meerlo et al. 1997). Beyond structural growth and morphology, the hormonal state can also affect energetic demands. Both estradiol and testosterone can, for instance, positively affect metabolic rate and energy expenditure (Buchanan et al. 2001; Gao et al. 2007). Similarly, glucocorticoid (e.g., cortisol or corticosterone) concentrations have been shown to be positively linked to metabolic rates in various bird species (Jimeno et al. 2017, 2020; Schwabl and Partecke 2020) and an interspecific analysis in mammals yielded the same result (Haase et al. 2016). Glucocorticoids are released in response to hypothalamic–pituitary–adrenal (HPA) axis activity and regulate a variety of metabolic processes (Vegiopoulos and Herzig 2007) and stress responses (Sapolsky et al. 2000). It is therefore not surprising that glucocorticoid concentrations can positively affect energetic demands.

In domestic guinea pigs (*Cavia aperea f. porcellus*) housed in same-sex groups, sex hormone concentrations increase during puberty and with sexual maturity (Schöpper et al. 2012a), but cortisol concentrations can also increase during this period (Schöpper et al. 2012b; Nemeth et al. 2018). This pattern has been shown to be more strongly pronounced in males than females and, moreover, increasing cortisol concentrations negatively affected body mass in males only (Nemeth et al. 2018). This might indicate that the establishment of social hierarchies during this phase of life could be more stressful in males, but this remains to be determined. The preliminary conclusion was that these sex-specific cortisol patterns and their effects on growth in adolescent guinea pigs indicated higher energetic requirements and correspondingly higher metabolic rate in males (Nemeth et al. 2018). This study therefore measured metabolic rate via oxygen (O_2_) consumption in male and female guinea pigs during adolescence until early adulthood in order to determine if it parallels a sex-specific pattern in cortisol concentrations during this period of life. Metabolic rate is usually positively affected by body mass and most studies include body mass in statistical analyses. Unfortunately, different approaches to (statistically) analyzing metabolic rate measurements may yield different results (Speakman 2013; Tschöp et al. 2012). In order to gain a more comprehensive insight into the energetic demands of adolescent guinea pigs, we (statistically) analyzed whole-body metabolic rate in ml O_2_/h, body mass-corrected metabolic rate in ml O_2_/h/kg, and body mass independent metabolic rate in ml O_2_/h statistically corrected for body mass.

We hypothesized that increasing cortisol concentrations with age in adolescent males are paralleled by a higher age-specific metabolic rate compared to females. Basal cortisol concentrations, however, have been found to be relatively unstable in male guinea pigs housed in mixed-sex groups during late adolescence and early adulthood, which was discussed as reflecting acute activity rates or food intake (Mutwill et al. 2021). Irrespective of any potential sex-specific changes in cortisol concentrations, instability on the individual level may also indicate fluctuations in energetic requirements. Nevertheless, metabolic rate is highly repeatable in vertebrates in the short- and long-term (Auer et al. 2016), and a study on pair-housed wild guinea pigs also showed repeatability in metabolic rate and even cortisol concentrations from juvenile to mature ages (Guenther et al. 2014). No information on metabolic rate and cortisol concentrations, as well as their repeatability, is available for domestic guinea pigs housed in single-sex groups. We therefore analyzed both parameters, as well as body mass, not only linked to sex and age but also regarding their repeatability. Different individual patterns in metabolic rate and cortisol concentrations during adolescence could also argue for a weak relationship between these two physiological parameters. Analyzing the repeatability in these physiological traits will provide valuable information on how stable they are on the individual level already early in life and during demanding growth processes.

## Methods

### Animals and housing

For this study, 12 male and 11 female domestic guinea pigs (*Cavia aperea f. porcellus*) were bred and kept at the department’s animal care facility. All animals were descendants of 9 adult females, which were mated with different males. They were born within 33 days of each other and their different natural fur color allowed individual identification. Pups were separated from their mothers at an age of 25 days (about 1 month before the study started) and housed in two same-sex groups, accordingly. Each enclosure measured 4.8 m^2^ and both were equally equipped with shelters and the floor covered with bedding. Daily provided food consisted of 30 g guinea pig pellets (Ssniff V2233, ssniff Spezialdiäten GmbH, Soest, Germany) per animal and 100 g hay per group. Water was available ad libitum in drinking bottles. The animals were kept in a temperature-controlled room (22 ± 2 °C; humidity: 50 ± 5%) with a light period from 7 a.m. to 7 p.m. Due to daily contact, all animals were familiar with humans.

### Study protocol

Measurements of body mass, saliva cortisol concentrations, and metabolic rate were carried out three times per animal during adolescence, at mean ages of 60, 90, and 180 days, with one individual being tested per day. This was possible because the animals were born over a time span of 33 days. However, animals from the same litter were tested on consecutive days, meaning at ages of 60, 120, and 180 (± 1) days.

Daily at 9 a.m., the focal animal was weighed on a standard kitchen balance and a saliva sample was collected for later cortisol analysis. For saliva collection, a cotton bud was inserted in the animal’s mouth for approximately 1 minute, then the sample was centrifuged (7378 g, 5 min) and stored at −20 °C. The focal animal was then placed in a transparent respiratory chamber to measure oxygen consumption as an indicator of metabolic rate for 2.5 h (for details see section “Metabolic rate measurement”). After the metabolic rate measurement, another saliva sample was collected from the focal animal before it was returned to its enclosure. While saliva cortisol concentrations before the metabolic rate measurement should indicate the (“basal”) stress load in relation to the social environment, concentrations afterwards were measured to identify stress responses to the measurement procedure.

### Metabolic rate measurement

Metabolic rate was measured using an open-circuit one-chamber respirometry system. The floor of the transparent respiratory chamber (8 l) containing the animal was covered with a thin layer of woodchip bedding to absorb excretions. An external pump pulled air through the chamber at a constant flow rate of 80 l/h. The air was dried over silica gel and subsequently measured by a portable oxygen analyzer (OxBox 4.1, constructed by T. Ruf and T. Paumann at the Research Institute of Wildlife Ecology, Department of Interdisciplinary Life Sciences, University of Veterinary Medicine Vienna, Austria; Schmid et al. 2000). Oxygen consumption in ml/h was measured in 30 s intervals over a period of 2.5 h, with 2 min reference readings of ambient air each 30 min. The oxygen analyzer and flow rate were calibrated prior to the study and before each measurement a zero calibration was conducted to compensate differences in pressure. Oxygen consumption was calculated in R 4.0.3 (R Core Team 2021) using a script provided with the OxBox. After excluding the first 10 min of measurement, corresponding to the time of the first full air exchange within the respiratory chamber, metabolic rate was calculated as the mean oxygen consumption over the remaining time to indicate the overall energy expenditure.

We decided to not analyze the resting metabolic rate, which is the lowest stable oxygen consumption over a certain period of time (e.g. 10 min), because of a high variation in oxygen consumption during the 30 s intervals (> 10%). This was probably because the animals did not remain calm inside the respiratory chamber for such a time span. The mean oxygen consumption analyzed here also includes higher values due to movements in the respiratory chamber and represents a mix of resting and active metabolic rate. Nevertheless, such movements should not come into play as strongly when considering the oxygen consumption over nearly 2.5 h compared to 10 min.

### Saliva cortisol analysis

Saliva cortisol concentrations were analyzed by biotin-strepdavidin enzyme-linked immunoassays using a cortisol-specific antibody and enzymes from the University of Veterinary Medicine, Vienna, Austria (for relevant cross-reactions see Palme and Möstl 1997), which has been validated for use in guinea pigs (Nemeth et al. 2016). Thawed saliva samples were diluted 1:50 using assay buffer. All analyses were done in duplicates. The intra- and interassay coefficients of variance were 14.93% and 13.77%, respectively. For further analyses, the individual cortisol response to the 2.5 h metabolic rate measurement was calculated by the natural logarithm of cortisol concentration after the measurement divided by concentration before the measurement: log (cortisol afterwards/cortisol before). This yielded values > 0 in case of an increase and values < 0 in case of a decrease in cortisol concentrations during the metabolic rate measurement. For one male, the amount of saliva was insufficient for analysis of cortisol concentrations after the metabolic rate measurement at an age of 180d, resulting in cortisol concentrations and the cortisol response of only eleven males for this age.

### Statistical analyses

Statistical analyses were carried out using R 4.0.3 (R Core Team 2021). All data were first analyzed by linear mixed models (LMMs) using library ‘nlme’ (Pinheiro et al. 2021) to determine differences in the respective variable between sexes and ages. All LMMs included “sex” (male, female), “age” (60, 120, 180 days), and their interaction as fixed factors. Individual and mother ID were included as random effects in each model to correct for repeated measurements and kinship, respectively (note: mother ID had no significant effect in any model). Models were selected based on the Akaike Information Criterion (AIC) through stepwise exclusion of non-relevant (non-significant) interactions and main effects. Only statistics for the remaining predictors are provided in the results section. For Bonferroni-corrected post-hoc analyses of selected models, the package ‘emmeans’ (Lenth 2020) was used.

The method of (statistically) analyzing metabolic rate (e.g. independent of body mass or dependent on body mass) can profoundly influence the results and their interpretation (Speakman 2013). In order to compare different approaches and to determine the effects of sex, age, and body mass in adolescent guinea pigs, metabolic rate was analyzed in (1) ml O_2_/h (whole-body), with “sex” and “age” as fixed factors, (2) ml O_2_/h/kg (body mass-corrected), with “sex” and “age” as fixed factors, and (3) ml O_2_/h with individual body mass as covariate (body mass-independent) additional to “sex” and “age” as fixed factors (body mass was included as main effect and no interactions with sex and age were allowed because of a low variance in body mass by age, which could have yielded poor validity in case of sex- and/or age-specific body mass effects).

Repeatability estimation was then performed regarding each parameter using library ‘rptR’ (Stoffel et al. 2017), which enables determining consistency/stability in individual differences. The repeatability associated with the random factor ‘individual ID’ was extracted from each analysis. As we expected effects of age and/or sex on each parameter, repeatability was always adjusted for both fixed factors.

Normal distribution and variance homogeneity of each model’s residuals were controlled by using Shapiro–Wilk normality tests and Levene tests for homogeneity of variance as well as by visual inspection of the residuals. Cortisol concentrations had to be transformed by applying the natural logarithm to meet the requirements, but raw data were used for representation of the results (note that no value was revealed as outlier after transformation). Significant levels were set at p ≤ 0.05. The whole dataset used for statistical analyses is provided as supplementary file (supplementary file 1).

## Results

### Body mass

Body mass was significantly affected by the interaction of sex and age (sex: F_1,13_ = 0.385, p = 0.546; age: F_3,63_ = 1353, p < 0.001; sex × age: F_3,63_ = 5.001, p = 0.004). Despite a similarly strong body mass gain, males showed a significantly higher body mass than females at an age of 60 days (60d: t = 2.965, p = 0.011, males: 577 ± 53 g, females: 546 ± 39 g), but no differences were detected at birth (0d: t = 0.621, p = 0.546, males: 96 ± 21 g, females: 106 ± 15 g) and afterwards (120d: t = 1.029, p = 0.322, males: 729 ± 70 g, females: 732 ± 45 g; 180d: t = 0.935, p = 0.367, males: 806 ± 45 g, females: 842 ± 42 g). A high repeatability in body mass was detected (R = 0.57, 95% CI: 0.36 – 0.75, p < 0.001).

### Saliva cortisol concentrations

**Fig. 1:**
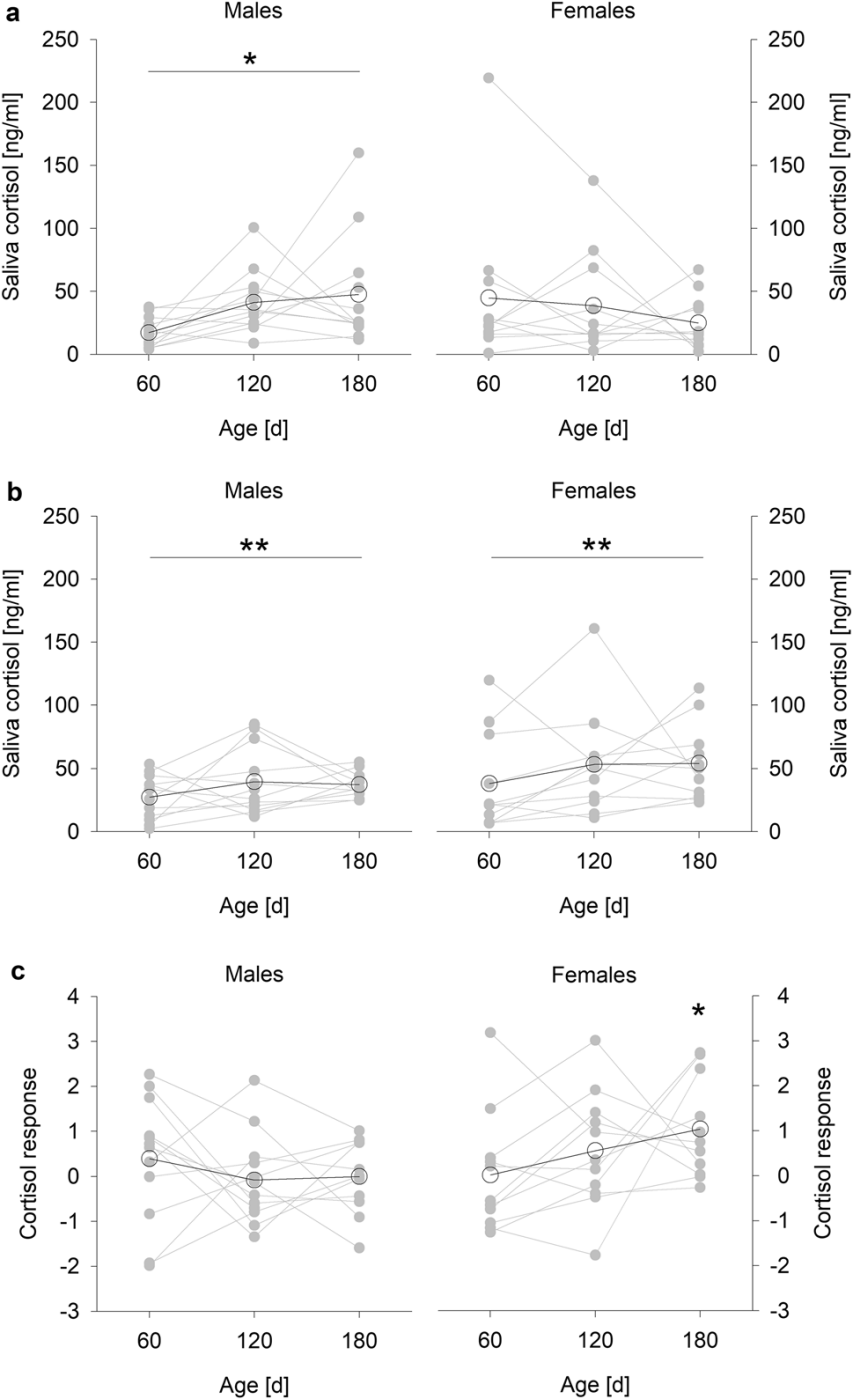
Saliva cortisol concentrations in male and female guinea pigs by age. Saliva cortisol concentrations (**a**) before the 2.5-h metabolic rate measurement and (**b**) after the 2.5-h metabolic rate measurement. (**a**, **b**) * p ≤ 0.05, ** p ≤ 0.01 (comparing ages). Note that cortisol concentrations had to be log-transformed for statistical analyses. (**c**) Cortisol response to the 2.5-h metabolic rate measurement = log (cortisol concentration after / cortisol concentrations before). * p ≤ 0.05 compared to 0, indicating no response at all. Open circles represent means per age, grey circles represent individual values

Saliva cortisol concentrations measured before the 2.5 h metabolic rate measurement (Fig. 1a) significantly increased with age in males (F_2,22_ = 6.812, p = 0.005) but not in females (F_2,20_ = 0.360, p = 0.702; full model statistics: sex: F_1,13_ = 1.955, p = 0.185; age: F_2,42_ = 0.460, p = 0.635; sex × age: F_2,42_ = 2.996, p = 0.061) and were not repeatable (R = 0.08, 95% CI: 0 – 0.39, p = 0.261; Fig. 1a).

After the metabolic rate measurement (Fig. 1b), saliva cortisol concentrations in general increased with age (F_2,43_ = 6.367, p = 0.004), while sex had no effect at all and was fully removed based on the AIC. A significant but not strongly pronounced repeatability was detected in these cortisol concentrations measured after the metabolic rate measurement (R = 0.26, 95% CI: 0.02 – 0.56, p = 0.020; Fig. 1b).

The saliva cortisol response (Fig. 1c) remained unaffected by sex, age, and their interaction (full model: sex: F_1,13_ = 0.581, p = 0.459, age: F_2,41_ = 2.056, p = 0.141, sex × age: F_2,41_ = 2.163, p = 0.128). However, females showed a significant cortisol response at an age of 180d (t = 3.102, p = 0.011), but no effects were detected at the remaining ages or in males (always p > 0.189). The cortisol response showed no repeatability (R = 0, 95% CI: 0 – 0.32, p = 1; Fig. 1c). Furthermore, saliva cortisol concentrations measured before the metabolic rate measurement had a general strong negative effect on the cortisol response (b = −0.927 ± 0.095; R^2^ = 0.58, p < 0.001).

### Metabolic rate

**Fig. 2:**
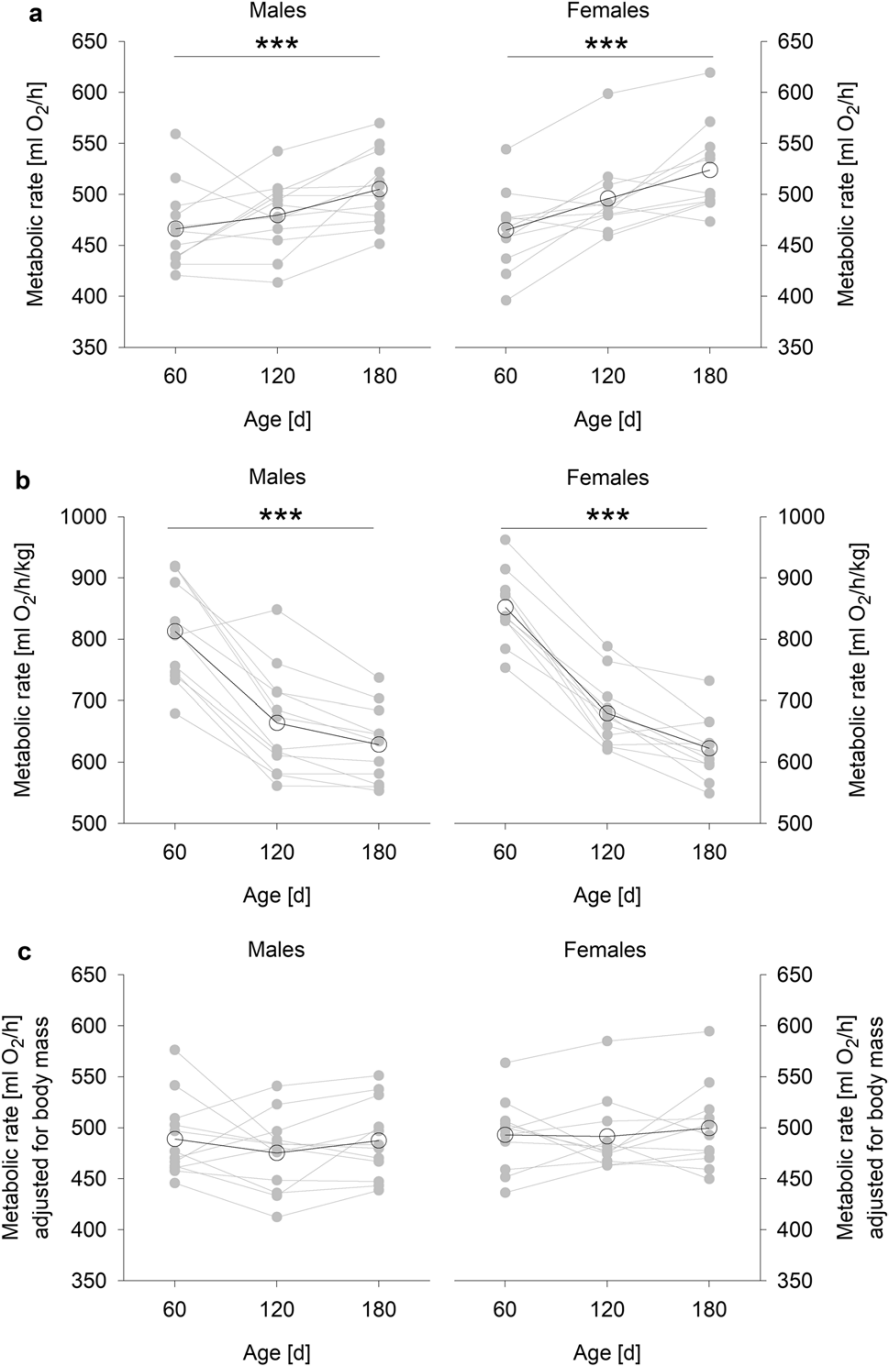
Metabolic rate (mean O_2_ consumption over 2.5 h) in male and female guinea pigs by age. (**a**) Whole-body metabolic rate (ml O_2_/h). (**b**) Body mass-corrected metabolic rate (ml O_2_/h/kg). (**c**) Body mass-independent metabolic rate (ml O_2_/h, statistically adjusted for body mass). *** p ≤ 0.001 (comparing ages). Open circles represent means per age, grey circles represent individual values

**Fig. 3:**
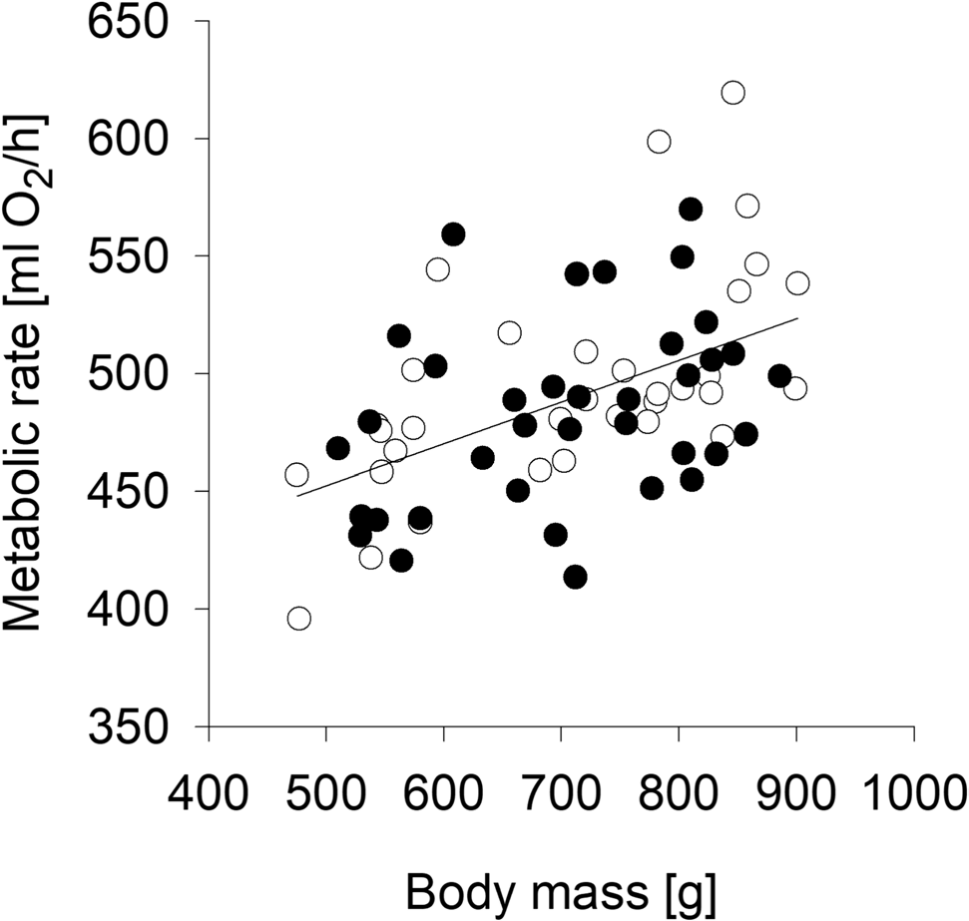
Main effect of body mass on metabolic rate (ml O_2_/h) based on the analysis of body-mass independent metabolic rate (R^2^ = 0.246, p < 0.001). Black circles represent males, open circles represent females

Whole-body metabolic rate (ml O_2_/h; Fig. 2a) increased with age (age: F_2,44_ = 19.815, p < 0.001), body mass-corrected metabolic rate (ml O_2_/h/kg; Fig. 2b) decreased with age (age: F_2,44_ = 179, p < 0.001), and the body mass-independent metabolic rate (ml O_2_/h, statistically adjusted for body mass; Fig. 2c) remained unaffected by age, but was positively affected by individual body mass (b = 0.188 ± 0.026, R^2^ = 0.246, p < 0.001; Fig. 3). No metabolic rate analysis revealed a significant sex difference (sex was removed based on the AIC); a slightly higher body mass corrected metabolic rate in females compared to males missed significance (F_1,13_ = 3.416, p = 0.088).

A highly significant repeatability in metabolic rate was found for each analysis, although repeatability was more strongly pronounced under consideration of body mass (whole-body metabolic rate in ml O_2_/h: R = 0.54, 95% CI: 0.31 – 0.76, p < 0.001; body mass-corrected metabolic rate in ml O_2_/h/kg: R = 0.69, 95% CI: 0.48 – 0.85, p < 0.001; body mass-independent metabolic rate in ml O_2_/h statistically adjusted for body mass: R = 0.61, 95% CI: 0.37 – 0.80, p < 0.001; Fig. 2a–c).

## Discussion

This study investigated the metabolic rate and saliva cortisol concentrations in adolescent guinea pigs, focusing on the effects of sex and age as well as repeatability and involving three different approaches of metabolic rate calculations and analyses. Adolescence is characterized by growth, changes in hormone balance, behavioral traits, and metabolic processes (Spear 2000), all of which may affect energetic demands during this period of life. Guinea pigs usually show the highest body mass gain during the first 60 days of life, which flattens at an age of approximately 100 to 120 days. Male cortisol concentrations in saliva can increase at this age, while female concentrations were shown to remain constant (Nemeth et al. 2018). Our study confirmed these patterns, supporting a sex-specific change in HPA-axis activity in adolescent guinea pigs housed in same-sex groups. Males housed in mixed-age/sex groups, for instance, did not show a pronounced change in plasma cortisol concentrations during adolescence (Hennessy et al. 2006; Mutwill et al. 2021). As older and heavier males are usually the dominant ones under mixed conditions (Mutwill et al. 2019), competition for social dominance might be higher and more stressful in a same-age/sex condition, because all males become mature and start to compete at the same time. Both receiving and initiating aggressive and/or dominant behavior can be linked to an acute increase in cortisol concentrations in guinea pigs, but such effects did not explain the age-dependent increase in cortisol concentrations in a previous study (Nemeth et al. 2020). Increasing cortisol concentrations with age in adolescent males may therefore reflect the social environment rather than being the result of social interaction rates, but also developmental processes such as testicular development and increasing testosterone concentrations have been found to affect cortisol concentrations during this period (Nemeth et al. 2018).

Nevertheless, none of the metabolic rate calculations and analyses performed in this study showed an expected sex-specific pattern by age as detected in cortisol concentrations. Accordingly, sex-specific changes in saliva cortisol concentrations during adolescence probably do not reflect different energetic demands in guinea pigs housed in same-sex groups. Studies showing positive relationships between glucocorticoid concentrations and metabolic rates in various species were based on high numbers of individuals and/or used profound stressors (see e.g., Brillon et al. 1995; Jimeno et al. 2018; Schwabl and Partecke 2020). Correlative approaches would have been inappropriate here because of the small number of animals involving both sexes, but the initial conclusion was that sexually different cortisol concentrations with age would be effective enough to be reflected in metabolic rate. The fact that metabolic rate did not differ between males and females in this study could be simply due to small “differences” in cortisol concentrations comparing sexes and ages and a high variation in individual cortisol concentrations. Other studies, however, which found no correlations between glucocorticoids and metabolic rates, suggest independent adjustment of these physiological traits (Buehler et al. 2012; Francis et al. 2018), but certain confounding factors must also be taken into account. In this context, note that the food provided in this study was limited to 30 g per animal and day, which was done to create standardized conditions for both sexes and all ages regarding metabolic rate measurements per se. Importantly, metabolic rate could have changed considerably in relation to cortisol concentrations during adolescence if growth rates and the available energy would not have been limited by this feeding regime. A study on wild guinea pigs found a negative correlation between cortisol concentrations and metabolic rate (Guenther et al. 2014), which the authors discussed as reflecting limited food availability. Under such a situation, energetically demanding behaviors or physiological processes (such as stress responses indicated by elevated cortisol concentrations) may limit the energy available for maintaining metabolic rate (Guenther et al. 2014; Careau and Garland Jr 2012). If such an effect limited metabolic rate in males despite increasing cortisol concentrations with age remains speculative and calls for further investigation.

As no uniform mode of metabolic rate measurement is available in the literature, three different approaches were used to analyze and compare metabolic rate. Analysis of whole-body metabolic rate in ml O_2_/h showed that the animals had higher total metabolic costs with increasing age and, correspondingly, body mass. During growth, the metabolic costs of synthesizing new tissues rise and larger digestive organs must be covered (Vézina et al. 2009). This was clearly equally pronounced in the male and female guinea pigs investigated here. Both sexes showed a similar body mass gain, although males were heavier at 60 days. This was probably because the animals were able to cope better with the limited amounts of food at a younger age until the limited food availability erased this difference at 120 days. Nonetheless, even the higher body mass of males aged 60 days was not related to a sex difference in whole-body metabolic rate. As body mass strongly affects metabolic rate, most studies include it in the analyses, simply by dividing oxygen consumption by body mass (reviewed by Tschöp et al. 2012). Our results reflect the effect of relating metabolic rate to body mass: whole-body metabolic rate in ml O_2_/h increased and body mass-corrected metabolic rate in ml O_2_/h/kg decreased with age. This is because the mass-specific metabolic costs are highest during periods of fastest development and drop by the end of structural growth. The problem with this approach is that the data can be distorted due to overcompensation of metabolic rate in larger and heavier individuals (Müller et al. 2021). Such an effect could have resulted in the slightly but not significantly higher body mass-corrected metabolic rate in females compared to males in this study, an effect that was mainly linked to the age of 60 days when males were heavier than females. A higher metabolic rate in females would contradict previous findings that implied higher metabolic rates in males or equal metabolic rates compared to female individuals when adjusting for body mass and/or body conditions (Careau et al. 2013; Guenther et al. 2014; Jimeno et al. 2018; Ferraro et al. 1992). An approach to overcome the problem of overcompensation is to adjust metabolic rate in ml O_2_/h statistically for body mass, for instance by analysis of covariance (Tschöp et al. 2012; Speakman 2013). This method corrects individual metabolic rate measurements to the same body mass level, thereby removing its influence. Including body mass as covariate in the LMM revealed that it generally had a positive effect on metabolic rate but that all sex- and age-effects were eliminated, yielding the body mass-independent metabolic rate. Although no analysis yielded a different metabolic rate in males and females (or a sex-specific change in metabolic rate with age), applying and comparing the three metabolic rate calculations and analyses provides useful information on how body mass or age in growing individuals contributes to metabolic rate.

The different patterns for cortisol concentrations and metabolic rate do not argue for a close relationship between the two. We partly explain this by the performed repeatability analysis and the cortisol concentrations after the metabolic rate measurement. Similarly to the results presented here, other studies on male and female guinea pigs showed low (or even no) repeatability in basal cortisol concentrations but a high repeatability in cortisol concentrations after a 2 h social separation stressor (Mutwill et al. 2021; Rystrom et al. 2022). The authors discussed this as effects of activity patterns, which can cause short-term fluctuation in “basal” cortisol concentrations. Individual differences in activity rates or social interactions may also explain the high within- and between-individual variance in cortisol concentrations as can be seen in the data presented here. Cortisol concentrations under stress probably reflect the maximum HPA-axis activity an individual can afford and can be assumed to be independent of such effects. Stress responses (e.g., the relative change in cortisol concentrations) can be limited by increased glucocorticoid concentrations (Romero et al. 2009), as has also been shown in Guinea pigs (Lürzel et al. 2010; Nemeth et al. 2021) and in the present study by the negative effect of initial cortisol concentrations on the cortisol response to the metabolic rate measurement. The cortisol response can therefore be interpreted as the result of the cortisol concentrations measured before the measurement and, similarly, revealed a very high variation. Accordingly, no repeatability in the cortisol response was found. A total time of 2.5 h in the respiratory chamber is potentially stressful and was assumed to result in high cortisol responses, but this was found only in females aged 180d, when initial cortisol concentrations were lowest. Cortisol concentrations before the measurement at the remaining ages and in males throughout may point to certain stress loads linked to the social environment, thereby not reflecting sex- and age-specific “baseline” values, and could have limited individual stress responses to the metabolic rate measurement. Cortisol concentrations in saliva have been shown to reflect those in plasma under basal and stimulated conditions and can be considered to adequately indicate stress responses in guinea pigs (Nemeth et al 2016). However, in contrast to cortisol concentrations, body mass was highly repeatable, indicating that body mass at birth determines body mass gain. Considering that body mass strongly determines metabolic rate, it is less surprising that metabolic rate was repeatable irrespective of the measured cortisol concentrations. This agrees with findings in wild guinea pigs, where the metabolic rate was repeatable from juvenile to mature phases of life (Guenther et al. 2014). The latter study also reported repeatability in plasma cortisol concentrations, but its experimental design and different housing and rearing conditions hinder a direct comparison to our results. Although the relationships on the individual level require closer examination, we suggest that different patterns in cortisol concentrations and metabolic rate on the individual level affect their connection or relationship. Nevertheless, while cortisol concentrations can be acutely affected by the social environment in several ways, the overall metabolic rate and hence energy expenditure is clearly very stable throughout adolescence.

We expected increasing cortisol concentrations in males with age to be paralleled by higher energetic demands compared to females, but this was not supported by our metabolic rate measurements. The role of glucocorticoids in energetic demands requires further investigation, because effects on metabolic rate have not been well-studied, neither inter- nor intraspecifically, as has also been critically assessed by others (Francis et al. 2018). Although the present study was not designed to test a direct correlation between cortisol concentrations and metabolic rate in guinea pigs, the results suggest that strong fluctuations in cortisol concentrations could affect their relationship to metabolic rate. The three different metabolic rate calculations and analyses preformed here are a step forward for future metabolic rate analyses in guinea pigs and can provide useful information with regard to other physiological influences, such as of cortisol concentrations.

## Supplementary Information

Below is the link to the electronic supplementary material.

Supplementary file1 (XLSX 15 KB)

## Data Availability

The dataset generated and analyzed during the current study is provided as supplementary file.
